# Genome Sequences of Three *Brucella canis* Strains Isolated from Humans and a Dog

**DOI:** 10.1128/genomeA.01688-16

**Published:** 2017-02-23

**Authors:** Marcus Vinicius Canário Viana, Alice Rebecca Wattam, Dhwani Govil Batra, Sébastien Boisvert, Thomas Scott Brettin, Michael Frace, Fangfang Xia, Vasco Azevedo, Rebekah Tiller, Alex R. Hoffmaster

**Affiliations:** a Laboratório de Genética Celular e Molecular, Universidade Federal de Minas Gerais, Belo Horizonte, Minas Gerais, Brazil; b Biocomplexity Institute of Virginia Tech, Virginia Tech University, Blacksburg, Virginia, USA; c Centers for Disease Control and Prevention, Atlanta, Georgia, USA; d Gydle, Inc., Québec, Canada; e Computing, Environment and Life Sciences, Argonne National Laboratory, Argonne, Illinois, USA; f Computation Institute, University of Chicago, Chicago, Illinois, USA; g Biotechnology Core Facility Branch, National Center for Emerging and Zoonotic Infectious Diseases, Centers for Disease Control and Prevention, Atlanta, Georgia, USA; h Mathematics and Computer Science Division, Argonne National Laboratory, Argonne, Illinois, USA; i Bacterial Special Pathogens Branch, Division of High-Consequence Pathogens and Pathology, Centers for Disease Control and Prevention, Atlanta, Georgia, USA

## Abstract

*Brucella canis* is a facultative intracellular pathogen that preferentially infects members of the Canidae family. Here, we report the genome sequencing of two *Brucella canis* strains isolated from humans and one isolated from a dog host.

## GENOME ANNOUNCEMENT

Brucellosis is a zoonotic disease caused by *Brucella* species that infect a diverse array of land and aquatic mammals, with humans as an accidental host (1). It is the most common zoonosis worldwide and induces an often chronic and incapacitating disease in humans, with low mortality (2). *Brucella* are stealth pathogens that have adapted to cause long-term chronic infections in their hosts (3). They are Gram-negative coccobacilli that live intracellularly within vertebrate hosts. *Brucella canis* is named due to its preference for hosts that belong to species of Canidae family (4), which includes dogs, coyotes, wolves, and foxes. Clinical signs of canine brucellosis range from asymptomatic infections to abortion and testicular atrophy. It is sexually transmitted, or by contact with aborted fetuses, food, or from an environment that has been contaminated by aborted material or excreta (5). *B. canis* infrequently infects humans, and the reported cases from such infection are usually described as mild (5).

Herein, we report the sequencing of three *B. canis* genomes. Strain 2009004498 was isolated from a human in Louisiana, USA, in January 2009. Strain 2009013648 was isolated from a human host in Arizona, USA, in April 2009. Strain 2010009751 was isolated from a dog (*Canis lupus familiaris*) in Massachusetts, USA, in January 2010.

The isolates were typed as *B. canis* sequence type 20 (ST20) by multilocus sequence typing (MLST) (6) and clustered with *B. canis* strains by multiple-locus variable-number tandem-repeat analysis (MLVA) (7). The genomes were sequenced using Roche 454 platform. The reads were checked for quality by FastQC 0.11.4 (http://www.bioinformatics.babraham.ac.uk/projects/fastqc/) and assembled by Newbler version 2.9 using a *de novo* strategy. Scaffolding was performed by CONTIGuator 2.7 (8), using *B. canis* ATCC 23365 (accession numbers CP000872.1 and CP000873.1) as reference. The gaps were closed by mapping the sequencing reads of each genome to *B. canis* ATCC 23365 and by extracting the consensus sequences (9) using CLC Genomics Workbench 6.5 (Qiagen, USA). A consistent automatic annotation by was generated by RASTtk (10) at the PATRIC bioinformatics resource center (11, 12).

Strain 2009004498 had 178,205 415,512 sequencing reads assembled in 31 contigs. The complete genome has two chromosomes, with a total of 3,307,321 bp, an average G+C content of 57.25%, 21.41× mean sequence coverage depth, 3,312 coding sequences (CDSs), 55 tRNA genes, and nine rRNA genes.

Strain 2009013648 had 397,813 sequencing reads assembled in 40 contigs. The draft genome has a chromosome I with one gap of 425 bp and a completely assembled chromosome II. The genome has a total of 3,313,885 bp, an average G+C content of 57.23%, 48.35× mean sequence coverage depth, 3,319 CDSs, 55 tRNA genes, and nine rRNA genes.

Strain 2010009751 had 415,512 sequencing reads assembled in 31 contigs. The complete genome has two chromosomes with a total of 3,277,586 bp, an average G+C content of 57.24%, 48.13× mean sequence coverage depth, 3,289 CDSs, 55 tRNA genes, and nine rRNA genes.

### Accession number(s).

The genome sequences of the strains have been deposited in GenBank under the accession numbers CP016973 and CP016974 for *B. canis* 2009004498, CP016975 and CP016976 for *B. canis* 2009013648, and CP016977 and CP016978 for *B. canis* 2010009751.
